# Prospecting Environmental Mycobacteria: Combined Molecular Approaches Reveal Unprecedented Diversity

**DOI:** 10.1371/journal.pone.0068648

**Published:** 2013-07-18

**Authors:** Alessandra Pontiroli, Tanya T. Khera, Brian B. Oakley, Sam Mason, Scot E. Dowd, Emma R. Travis, Girum Erenso, Abraham Aseffa, Orin Courtenay, Elizabeth M. H. Wellington

**Affiliations:** 1 School of Life Sciences, University of Warwick, Coventry, United Kingdom; 2 USDA Agricultural Research Service, RBRRC, Athens, Georgia, United States of America; 3 Molecular Research LP (MR DNA), Shallowater, Texas, United States of America; 4 Armauer Hansen Research Institute (AHRI), Addis Ababa, Ethiopia; National Institute of Infectious Diseases, Japan

## Abstract

**Background:**

Environmental mycobacteria (EM) include species commonly found in various terrestrial and aquatic environments, encompassing animal and human pathogens in addition to saprophytes. Approximately 150 EM species can be separated into fast and slow growers based on sequence and copy number differences of their 16S rRNA genes. Cultivation methods are not appropriate for diversity studies; few studies have investigated EM diversity in soil despite their importance as potential reservoirs of pathogens and their hypothesized role in masking or blocking *M. bovis* BCG vaccine.

**Methods:**

We report here the development, optimization and validation of molecular assays targeting the 16S rRNA gene to assess diversity and prevalence of fast and slow growing EM in representative soils from semi tropical and temperate areas. New primer sets were designed also to target uniquely slow growing mycobacteria and used with PCR-DGGE, tag-encoded Titanium amplicon pyrosequencing and quantitative PCR.

**Results:**

PCR-DGGE and pyrosequencing provided a consensus of EM diversity; for example, a high abundance of pyrosequencing reads and DGGE bands corresponded to *M. moriokaense*, *M. colombiense* and *M. riyadhense*. As expected pyrosequencing provided more comprehensive information; additional prevalent species included *M. chlorophenolicum, M. neglectum, M. gordonae, M. aemonae*. Prevalence of the total *Mycobacterium* genus in the soil samples ranged from 2.3×10^7^ to 2.7×10^8^ gene targets g^−1^; slow growers prevalence from 2.9×10^5^ to 1.2×10^7^ cells g^−1^.

**Conclusions:**

This combined molecular approach enabled an unprecedented qualitative and quantitative assessment of EM across soil samples. Good concordance was found between methods and the bioinformatics analysis was validated by random resampling. Sequences from most pathogenic groups associated with slow growth were identified *in extenso* in all soils tested with a specific assay, allowing to unmask them from the Mycobacterium whole genus, in which, as minority members, they would have remained undetected.

## Introduction

Environmental mycobacteria (EM), also known as nontuberculous, opportunistic or atypical mycobacteria, belong to the genus *Mycobacterium* but are generally considered distinct from the *M. tuberculosis* complex, especially in terms of the impact on public health [1]. EM comprise both saprophytic species and a number of pathogens that have been identified as causative agents of a wide range of diseases such as pulmonary, cutaneous and disseminated diseases, posing a particular risk for those that are immunocompromised [2]–[4]. EM species are characteristically and phylogenetically separated on the basis of growth into fast growing and slow growing mycobacteria [5], with pathogenicity predominantly correlated with slow growth [6]. The number of newly identified Mycobacteria species from the environment or in clinical settings has risen from about 100 species to 157 in the last decade [7], [8] (and http://www.bacterio.cict.fr/m/mycobacterium.html); of these a third are potential human and animal pathogens. In addition, evidence suggests that exposure to EM interferes with the efficacy of the BCG vaccination against adult pulmonary tuberculosis [9].

EM are ubiquitous in the environment, having been isolated from a variety of sources including natural waters, drinking water distribution systems [10]–[12], biofilms, hot tubs and spas, peat, acid brown swamps, potting soils [13] reed beds [14] and acidic boreal forest soils [15]–[18]. They have also been isolated from dust, milk and from the salivary glands of insects [19]–[21]. To date, EM diversity and prevalence in the environment has been mainly assessed using cultivation, which is an insensitive method particularly for selective groups. Therefore, to date, few studies have reported slow-growing EM in soil isolation studies [15], [22], [23]. Previously, we reported one of the first analysis of diversity of slow growing mycobacteria in soil [24] and subsequently developed a suite of techniques for reliable extraction and detection of *M. bovis* in soil. Direct diversity analysis of EM in soil has focused on 16S rRNA genes using PCR-DGGE, clone libraries and T-RFLP [24]–[30]. These studies aimed either at the whole genus or at slow growers, but through targeting the whole genus, they risk underrepresentation of the low prevalence slow growers which have a single *rrn* operon [31] copy. To address this issue we report here the development of a combined approach to determine the diversity of EM which includes slow growers and hence the *M. tuberculosis* and the *M. avium* complex too, using the 16S rRNA gene as target. Here a method was devised which exploited a particular signature in the Mycobacterium genus 16S rRNA gene, known as the long helix 18 [6], which is associated with pathogenicity and is harboured by the majority of slow growers known to date. Our aims were i) to perform a bioinformatics analysis with a customized approach for EM detection using tag-encoded Titanium FLX amplicon pyrosequencing ([32]), ii) to compare deep sequencing with community profiling using PCR-DGGE and iii) to determine if ratios of fast to slow growing EM correlate with their distribution in selected soil environments via quantitative PCR (qPCR).

## Materials and Methods

### Bacterial strains and growth conditions

The strains used in this study are listed in Table 1. All species were stored at −80°C as glycerol stocks. The stocks were resuscitated on Middlebrook 7H10 selective agar plates (BD, Oxford, UK) and grown at 37°C until colony formation. A single colony was then inoculated onto Middlebrook Broth 7H9 (BD, Oxford, UK) with constant shaking at 150 rpm. For *Mycobacterium avium subsp. paratuberculosis* a supplement of ferric mycobactin J (Allied Monitor, Fayette, Missouri, USA) was added to both the media and broth following the manufacturer instructions.

**Table 1:** Strains used in this study.

Species	Strain	Growth fashion
*Mycobacterium abscessus*	DSM 44196T*	F
*Mycobacterium agri*	****	F
*Mycobacterium aichiense*	****	F
*Mycobacterium aurum*	ATCC 23366***	F
*Mycobacterium avium subsp. avium*	DSM 44156T*	S
*Mycobacterium avium subsp. paratuberculosis*	DSM 44133T*	S
*Mycobacterium bovis BCG*	Pasteur	S
*Mycobacterium chubuense*	****	S
*Mycobacterium duvalii*	****	F
*Mycobacterium fortuitum*	ATCC 14468***	F
*Mycobacterium fortuitum*	DSM 46621T*	F
*Mycobacterium gilvum*	****	F
*Mycobacterium gordonae*	ATCC 14470***	S
*Mycobacterium intracellulare*	DSM 43223T*	S
*Mycobacterium kansasii*	DSM 44162T*	S
*Mycobacterium marinum*	DSM 44344T*	S
*Mycobacterium nonchromogenicum*	ATCC 19530***	S
*Mycobacterium obuense*	****	F
*Mycobacterium peregrinum*	****	F
*Mycobacterium phlei*	ATCC 354***	F
*Mycobacterium scrofulaceum*	DSM 43992T*	S
*Mycobacterium smegmatis*	ATCC 13578***	F
*Mycobacterium vaccae*	DSM 43292T*	F
*Mycobacterium xenopi*	DSM 43995T*	S
*Streptomyces coelicolor MX*	EMW lab	-

* Deutsche Sammlung von Mikroorganismen und Zellkulturen GmbH Mascheroder Weg 1b, 38124 Braunschweig, Germany *** American Type Culture Collection (ATCC), P.O. Box 1549, Manassas, VA 20108, USA****Supplied by John Magee, Regional Centre for Mycobacteriology, Newcastle, UK.^ 1^ Growth fashion: F =  fast; S =  slow.

### Soil Samples

A Material Transfer Agreement was made with the Institute of Biodiversity and Conservation, Ethiopia, for export of soils. A DEFRA Licence to import, move and keep prohibited soil (Licence No. PHL 208/6138) was issued. The study was carried out on private land and the owners of the land gave permission to conduct the study on the sites. Samples were collected in duplicate from the upper layer (0–5 cm) of sites in and around the market town of Mehal Meda, Ethiopia (soils 1–4) and from Cryfield field, Cryfield Farm, Warwick University, Warwick, United Kingdom [33]. Soil sample 1 (1108), described as very compacted and dry soil was collected from the ground used for cattle and goods in the market place of Mehal Meda town (10°31′N, 39°66′E). Soil sample 2 (1109), was collected from the compacted soil floor at the entrance to a house near Mehal Meda (10°33′N, 39°74′E). Soil sample 3 (1110), was collected from a ploughed field in Mezezo (9°97′N 39°74′E). Soil sample 4 (1111), was collected from a house yard on a roadside in Sembo (9°42′N 39°35′E). Soil samples were collected in October 2008 during the Ethiopian dry season. The soil sample 5 from Cryfield, (52°36′N 1°56′E) was collected in August 2008. Samples were stored at room temperature and were thoroughly mixed before extraction. The sampling was preliminary to a subsequent field survey throughout varying agroclimatic regions in Ethiopia.

### DNA Extraction

Genomic DNA from the various reference strains of Mycobacteria was extracted using the DNeasy Blood & Tissue Kit (QIAGEN, Ltd., Crawley, UK) following manufacturer's instructions. Total soil DNA was extracted from soil samples (0.5 g±0.2 g) with the FastDNA Spin Kit (MP Biomedicals, OH, USA) as per manufacturer’s instructions.

### Primer design

The primers used in this study are shown in Table 2. Primers previously published by our group (Young et al., 2005) were initially assessed as their specificity had been proven. These primers targeted two different sequences in the 16S rRNA gene, encompassing the regions V2–V4 (termed *Mycobacterium* genus specific JSY1SF/R primer set) and V3–V6 which contains the long helix 18 insertion (of a variable size of 28 to 31 bp), distinctively present in a large subgroup of slow growing mycobacteria [6], [24], termed JSY16Sslow primer set. Preliminary work showed that the length of the amplicons generated by the JSY16Sslow primer set (605 bp) was problematic for DGGE, therefore, a new primer set APTK16S was designed and customized for the three methods. The new primer set was designed with the PrimerBlast software (http://www.ncbi.nlm.nih.gov/tools/primer-blast/primerinfo.html); including the V3 region and specifically targeting the helix 18 insertion and upstream regions, globally, V1–V3 (termed APTK16S). Theoretically, for the regions targeted in this study, at a species differentiation similarity cutoff of 97%, a minimum difference of 12 bp from each strain is expected in order to distinguish a species, based on the minimal sequence length of 405 bp amplified by the primers. Fifty-one selected reference strains including 21 slow growers (listed in Table S3 and Table S4 in File S1) were analyzed with a similarity matrix and differed from each other by 7–74 bp in the V1–V3 region and by 4–76 bp in the V2–V4 region.

**Table 2:** Primers used to target mycobacteria for diversity analysis.

Primer Name	Sequence	Target group	Amplicon size and 16S rRNA gene regions	Reference
1. pA	5'-AGAGTTGTTTGATCCTGGCTCAG-3'	Eubacteria	1542 bp – V1–V9	(Edwards et al., 1989)
2. pH	5'-AAGGAGGTGATCCAGCCGCA-3'			
3. JSY16SF	5'-TGGGAAACTGGGAAACTGGGTCTAATA-3'	Mycobacterium genus	465 bp – V2–V4	(Young et al., 2005)
4. JSY16SR	5'-CCCGCACGCCCAAGTTAAGCTGTGAG-3'			
5. JSY16SSlowF	5'-CGACGAAAGGTCCGGGTTCTCTCGGATTGAC-3'	Slow growing Mycobacteria^1^	605 bp – V3–V6	(Young et al., 2005)
6. JSY16SSlowR*	5'-GCCATGCACCACCTGCACACAGGCCCAC-3'			
7. APTK16SF	5'-GCTTAACACATGCAAGTCGAACGGAAAGG-3'	Slow growing Mycobacteria^1^	405–438 bp – V1–V3	This paper
8. APTK16SR*	5'-GTCAATCCGAGAGAACCCGGACCTTCGTCG-3'			
9. APmycoF	5'-GCAAGCCTGATGCAGCGACG-3'	Mycobacterium genus	232 bp – V2 (9 and 4)	This paper
10. APslowF	5'-GGGATAAGCCTGGGAAACTGGGTCTAATAC-3'	Slow growing Mycobacteria^1^	332 bp – V1 (10 and 8)	
11. APTK16SF-Pyro	5'-GCTTAACACATGCAAGTCGAACG-3'	Slow growing Mycobacteria^1^	405–438 bp – V1–V3	This paper
12. APTK16SR-Pyro	5'-GTCAATCCGAGAGAACCCGGAC-3'			

* to these primers a GC clamp was added for DGGE; ^1^ those that possess the long helix 18 (Leclerc et al., 2003).

As qPCR intrinsically requires shorter amplicons, we used the same software to design a suitable forward primer APslowF for the slow grower specific qPCR assay and a suitable forward primer APmycoF for the genus specific qPCR assay to be used with JSY16SR.

For DGGE, A GC clamp (5′CGCCCGCCGCGCGCGGCGGGCGGGGCGGGGGCACGGGGGG 3′) was added to the reverse primers JSY16SR and APTK16SR and used in PCR reactions.

### PCR and quantitative PCR conditions

In order to reliably generate PCR amplicons for DGGE, nesting was applied to all soil extracted DNA using the primer set pA and pH (Table 2) to first amplify the whole 16S rRNA gene [34]. Direct PCR attempts had failed to sufficiently produce amplicons in a standardized fashion for all samples. Reactions were performed in a 50 µl volume containing 1 µl of DNA, 25 µl of Promega PCR master mix (Promega Ltd., Southampton, UK), 1 µl (10 µmol) of each primer, 2.5 µl DMSO, 2 µl (10 mg/ml) Bovine Serum Albumine (BSA)(Sigma-Aldrich, Dorset, UK), and 17.5 µl of filter sterilized MonoQ water.

The PCR cycle was 94°C for 1 min, followed by 40 cycles of 94°C for 1 min, 62°C for 1 min, 72°C for 1.30 min and a single extension step of 72°C for 7 mins. Subsequently, specific 16S rRNA gene regions were amplified in the following manner: each 50 µl reaction contained 1 µl of DNA, 25 µl of Promega PCR master mix (Promega Ltd., Southampton, UK), 1 µl (1µmol) of each primer, 2.5 µl DMSO, 2 µl (10 mg/ml) Bovine Serum Albumine (BSA)(Sigma-Aldrich, Dorset, UK), and 17.5 µl of filter sterilized MonoQ water. The thermal protocol for the JSY16SF/R primer set was 94°C for 5 mins, followed by 35 cycles at 94°C, 55°C for 1 min, 65°C for 1 min, and finally a single extension step of 65°C for 5 mins. For the APTK16SF/R primer set it was 94°C for 5 min, followed by 35 cycles at 94°C for 1 min, 59.9°C for 1 min, 67°C for 1 min, and finally a single extension step of 67°C for 5 mins.

For qPCR, amplification reactions were set up and run in triplicate with ABI 7500 Fast Real-Time PCR System (Applied Biosystems Inc., CA, USA). For both the genus specific and the slow growers specific qPCR reactions, 12.5 μl of SYBR® Green PCR Master Mix (Applied Biosystems Inc, CA, USA) were mixed with 2.5 μl of a 0.45 µM primer Mastermix (made up from a stock of 10 µM of each primer), 0.25 μl BSA (10 mg/ml) (Sigma-Aldrich, Dorset, UK), and 4.75 μl of filter sterilized MonoQ water to which 1 μl of DNA template directly extracted from soil was added for each sample. Plates were analyzed with the auto threshold and a melting curve analysis was included. *Mycobacterium bovis* BCG genomic linear DNA standard dilutions were used to generate a standard curve for absolute quantification and were run in triplicate on each quantitative plate. They were converted to genomic equivalents [33] and ranged from 8.5×10^5^ copies/μl to 8.5 copies/μl. The thermal cycling protocol used was an initial step of 2 min at 50°C followed by 10 mins at 95°C, then by 40 cycles consisting of 20 secs at 95°C and 1 min at 60 C. The final dissociation step consisted of 15 secs at 95°C, 1 min at 60°C for and 15 secs at 95°C. As seen previously with a different assay (Pontiroli et al.[33]), the use of an inhibition control via an internal amplification control confirmed the lack of inhibitors with good amplification of DNA on DNA extracted from soils of varying characteristics with the FastDNA Spin Kit, and the yields of the extractions here were very comparable across all samples tested, so copy numbers were corrected only by subtracting the noise due to background fluorescence typically generated by SYBR® Green at the plateau stage of the Real Time PCR. For all reactions, the R^2^ values of the standard curve were at least 0.97 and the reaction efficiency varied between 90–100%. Estimates of the titer of the fast growers were obtained by subtracting the counts of the slow growers from the total cell counts and by dividing the number of the fast growers by two, considering the double copy of *rrn* operons of the latter.

### Denaturing gradient gel electrophoresis (DGGE)

DGGE was performed using the DCode Universal Detection System according to the manufacturer's instructions (Bio-Rad Laboratories, CA, USA). An 8% polyacrylamide gel (acrylamide-bis-acrylamide 37.5: 1) in 0.5 X TAE buffer was used. The denaturing gradient for the *Mycobacterium* genus specific JSY16S PCR products was 45%–55% where 100% denaturant corresponds to 7M Urea and 40% [vol/vol] deionized formamide (Muyzer *et al.,* 1993). The denaturant gradient for the slow growing specific APTK16S PCR products was 40%–60%. PCR products (22 µl) with 8 µl of Fermentas 6x DNA loading dye were loaded into each lane of the gel. Electrophoresis was carried out initially for 10 minutes at 150 V and subsequently for 960 mins at 60 V at 60°C in 7 litres of 0.5 X TAE buffer (40 mM Tris-acetate and 1 mM EDTA, pH 8.0). The gels were stained with Ethidium bromide (10 mg/ml) for 15 mins and destained with distilled water for 20 minutes and photographed (GeneFlash Gel Documentation System, Syngene, Cambridge, UK). Major bands were excised and then reamplified with either the JSY16S primer set or the APTK16S primer set without the GC clamp. The PCR products were purified using the QIAquick Gel Extraction Kit (QIAGEN, Ltd., Crawley, UK) according to the manufacturer's instructions, and then sequenced using an ABI PRISM 3130xl Genetic Analyser (Life Technologies Ltd, Paisley, United Kingdom). All sequences were aligned using ClustalW (http://www.ebi.ac.uk/Tools/msa/clustalw2) and compared with the nucleotide database in Genbank using BLASTn (http://www.ncbi.nih.gov/) and with Silva v.104 database using a standalone BLASTn (see below). In order to determine the limit of detection of the technique, i.e. to assess the varying analytical sensitivities obtained with the different primer sets, soil microcosms consisting of 0.5 g (±0.1) sterile soil microcosms were seeded in duplicate with a dilution series of *M. bovis* BCG cells which ranged from of 10^1^ to 10^6^ equivalents of genomic DNA. DNA was extracted and was amplified either directly with the JSY16S and APTK16S primer sets or after nesting, necessitating a preliminary amplification with pA and pH primer sets prior to the DGGE run, in order to determine the detection limit of the DGGE method. *M. bovis* BCG genomic DNA was used as control for an independent run in the electrophoresis.

### Pyrosequencing sample preparation, tag design and sequencing PCR

DNA samples were normalized to 100 ng/µl and 1 µl aliquots were used to amplify the genus and slow grower specific regions with customized primers (Table 2). Pyrosequencing was carried out using tag encoded FLX-Titanium amplicon pyrosequencing similar to that described previously [32] including the use of Titanium reagents and a mixture of Hot Start and HotStart high fidelity Taq polymerases as well as a one-step PCR, with 30 cycles of PCR to reduce chimera formation [35]. Pyrosequencing was performed at the Research and Testing Laboratory (Lubbock, TX) based upon RTL protocols (www.researchandtesting.com).

#### Pipeline for sequences analysis

A customised bioinformatics pipeline was developed to process sequences, using individual and shell scripts in a Linux environment. Sequences were trimmed in mothur [36] using the quality files with a 50 bp moving window at an average quality score cutoff of 35, and a maximum number of homopolymers below eight. Further processing as per recent recommendations [37]–[39] was done with Perl and Bioperl scripts which trimmed pyrosequencing tag sequences, screened for presence of the forward PCR primer sequence, and removed sequences with any ambiguous base calls. Based on expected amplicon sizes and sequence length frequency distributions of bacterial sequences in the Silva reference database (v.104), sequences were further limited to a range of 468–472 bp with JSY16S primer set and 420–424 bp for the amplicons obtained with APTK16S. Sequences which passed these screens were aligned to the reference dataset used for blastn using PyNAST [40] implemented in QIIME [41] and putative chimeric sequences were identified with ChimeraSlayer [42] following the authors' specifications, in order to identify and remove the chimeric sequences potentially generated by pyrosequencing. The proportion of chimeras detected was 5.66% for the genus data set (1544 intragenus chimeras) and 0.58% for the slow grower data set (59 intragenus and 4 intraorder chimeras).

Sequences which passed all the screens described above were compared with blastn run locally against the reference database described above derived from the Silva project (v.104) curated seed database [43]. For blastn, the highest ranked hit to this dataset was retained using an e-value of 10^−4^. The number of reads pre- and post quality control are shown in Table 3.

**Table 3:** Characteristics of the pyrosequenced libraries including number of reads pre and post quality control, OTU, EM counts as determined by qPCR, richness diversity indices and non parametric diversity estimators.

	number of sequences													
Libraries	initial	primer found	after trimming	OTU	OTU_R	Gene copies g^−1^ (±) SD	H'	H'_R	Simpson	Simpson_R	Chao1 (± SE)	Chao1_R(± SE)	ACE (± SE)	ACE _R(± SE)
	**GENUS**													
**1008**	17647	14718	8324	186	81	9.1×10^8^±6.9×10^7^	3.95	3.49	0.96	0.952	244.57±28.18	120±24,75	228.85±7.59	112,93±5,64
**1109**	14839	12975	7248	214	91	8.2×10^8^±3.3×10^8^	4.07	3.74	0.969	0.96	273.37±25.16	117,25±19,03	257.28±8.11	106,29±5,02
**1110**	6634	5909	1453	149	108	2.3×10^8^±4.4×10^7^	4.06	3.68	0.969	0.954	178.29±12.71	133,38±13,74	186.05±6.69	129,99±5,49
**1111**	5161	4623	1135	101	74	7.4×10^8^±2.7×10^6^	3.24	2.8	0.923	0.87	146.56±20.78	102,11±18,97	158.87±7.02	96,43±4,93
**Cryfield**	15997	13853	9098	170	76	2.7×10^8^±2.4×10^8^	3.86	3.25	0.962	0.934	201.06±15.71	91±9,69	198.96±6.87	94,25±4,73
*total*	**60278**	**52078**	**27258**	**314** *	**158** *									
	**SLOW**													
**1008**	2066	1851	1533	18	14	8.9×10^5^±1.6×10^5^	1.882	1.618	0.78	0.714	18±0	14±1,32	18±2	14,45±1,84
**1109**	5399	4991	4119	20	14	1.2×10^6^±4.8 ×10^5^	0.617	0.349	0.219	0.119	20.75±2.29	16,5±4,88	23.61±2.5	21,39±2,51
**1110**	3259	2956	1381	20	18	1.0×10^7^±2.3×10^6^	1.753	1.495	0.729	0.689	20.2±1.03	46±NaN	21.18±2.23	75,95±3,34
**1111**	5037	4647	1584	17	12	4.9×10^6^±1.3×10^6^	0.595	0.466	0.216	0.177	22±17.14	22±NaN	21.09±2.12	20,06±2,05
**Cryfield**	2952	2776	2353	18	13	2.9×10^5^±4.2×10^4^	1.734	1.533	0.771	0.731	19.5±7.19	13±0	22.74±2.44	13±1,75
*total*	**18713**	**17221**	**10970**	**33** *	**26** *									

* unique OTUs; NaN: not available; _R: random resampling; SD: standard deviation of the mean triplicate experiments.

#### Classification of metadata into OTU based on similarity to reference sequences

Filtered sequences were clustered into similarity-based OTUs (Operational Taxonomic Units) using a shell script which combined the two softwares CD-HIT-EST [44] and mothur version 1.5.0 [45] by using a series of scripts edited in Perl and R (http://www.r-project.org). Based on preliminary testing, four sequence similarity cutoffs for CD-HIT-EST (99, 97, 95, 93%), were set as default in the shell script.

### Random resampling

In order to take into account any potential bias resulting from different numbers of sequences among different soil samples, a script was developed in R to randomly select equal numbers of sequences from each site for each primer set; the resulting fasta files were used as input files for the above described processing pipeline. Output data was compared to non randomized results (Table 3) obtained with the clustering approach previously described.

### Statistical analysis

The cluster output files were automatically converted to tabular format with Perl scripts and input into mothur which generated similarity based distance matrices and computed derivative diversity estimates such as Shannon (H’) and Simpson and the nonparametric species richness estimators ACE and Chao1. Concomitantly, an automated summary analysis of OTU frequency distributions, including rarefaction curves and CCA was generated in R. Clustering analysis, principle component analysis, P test significance and Unifrac significance test were calculated using FastUniFrac. The χ2 test for OTU richness analysis was performed in Excel 2007 (Microsoft Co., USA).

### Phylogenetic assignment via standalone BLAST

Quality controlled sequences and representative sequences were compared to the SILVA SSU rRNA database (Release 104, 2010); we modified the Silva reference database to ensure the most accurate possible classification of Mycobacteria to the species level by adding all available Mycobacteria sequences (n = 899). Sequences were compared at first to the whole SSU-16S rRNA and then only to the Mycobacteria species from the customised database with BLASTn. For each query sequence, the best match was retained for sequences with at least 97% identity and minimum query coverage of 90%.

### Phylogenetic diversity analysis

Sequences were aligned against a template alignment containing 16S rRNA genes from Mycobacteria exported from the SILVA v.104 database using the align.seqs function in mothur v. 1.5.0 [45]. The aligned fasta file was then imported into ARB [46] and used for the construction of phylogenetic trees using maximum likelihood and neighbour-joining algorithms. Output files containing cluster related information and frequency distribution were retrieved from the pipeline and selected cluster representative sequences imported into ARB in order to compare their topology with the Mycobacteria database. MrBayes (http://mrbayes.sourceforge.net/index.php) was also used for phylogenetic reconstruction with default parameters except for a general time reversible (GTR) evolutionary model and gamma distributed rate variation across sites. The analysis was run for 2,000,000 generations, after which the convergence diagnostic, the average standard deviation of split frequencies, was <0.008. To assess the differences between soil samples using phylogenetic inferences, the FastUniFrac web interface was used [47], [48] which uses branch length and position to compare actual phylogenies to a null model of randomly permuted sites. Phylogenetic trees were built with ARB neighbour-joining method and the sample mapping file and the category mapping file were used as input for FastUniFrac analysis.

## Results

### Real Time quantification of EM

Successful, sensitive and specific qPCR was achieved with both primer sets, with the APTK16S primer set amplifying exclusively the slow growing mycobacteria in the panel tested as expected (Table 1).

EM communities were detected from bulk soil DNA extracted from five soils; counts were in the range of 2.9×10^5^ to 1.2×10^7^ cells g^−1^ of soil for slow growers and 2.3×10^7^ to 2.7×10^8^ gene targets g^−1^ of soil when the *Mycobacterium* genus was targeted (Table 3). The vast majority of fast growers have two copies of 16S rRNA genes compared to a single copy in slow growers ([31]), allowing estimation of fast grower titer of between 1.135×10^7^ and 1.29×10^8^ and of slow growers between 2.9×10^5^ to 1.2×10^7^ cells g^−1^ of soil. This reflects the fact that fast growers are typically more abundant and outcompete slow growers in the environment. In addition to the differences in copy number of 16S rRNA genes the V3–V6 amplicons may skew results giving an underestimation of the slow growing mycobacteria, especially as the amplification targets of the two groups are from different regions. Global counts measured with both primer sets were in the same range as those reported in the study of Jacobs et al. ([30]) where a Taqman assay targeting another portion of the 16S rRNA gene was used.

### Profiling bacterial communities by DGGE

**Figure 1:**
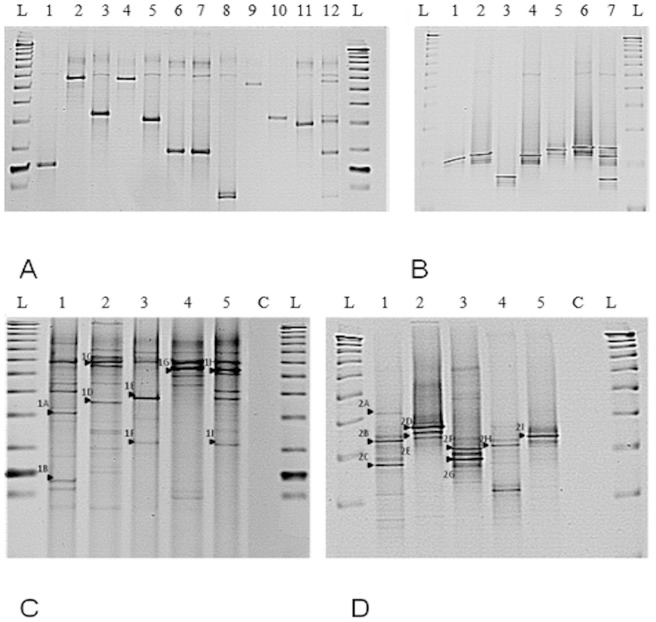
DGGE EM profiling using 16S rRNA gene PCR amplicons. (A) Profiles of individual EM type strains obtained using the *Mycobacterium* genus specific primer set JSY16S. L is the reference ladder (TrackIt™ 50 bp DNA Ladder, Invitrogen, Ltd., Paisley, UK), lanes 1–12 are, respectively: *M. smegmatis*, *M. aichiense*, *M. aurum*, *M. gilvum*, *M. phlei*, *M. agri*, *M. peregrinum*, *M. duvalii, M. abscessus*, *M. fortuitum, M. vaccae*and a mixture of equimolar quantities of the above listed EM species. (B) Profiles obtained using the slow mycobacteria specific primer set APTK16S. L is a reference ladder (TrackIt™ 50 bp DNA Ladder, Invitrogen, Ltd., Paisley, UK), lanes 1–7 are, respectively: *M. intracellulare, M. marinum*, *M. kansasii*, *M. xenopi*, *M. aviumparatuberculosis*, *M. bovis BCG* and a mixture of equimolar quantities of the above listed EM species. (C) EM soil community profiling using the *Mycobacterium* genus specific primers (JSY16S). L is a reference sizing ladder (TrackIt™ 50 bp DNA Ladder, Invitrogen, Ltd., Paisley, UK), C is the negative PCR control, samples 1 – 4 are the four Ethiopian soils (1108, 1109,1110, 1111) and 5 is Cryfield. The arrows (1A–1I) indicate the bands that were excised and sequenced (Table 4). (D) EM soil community profiling using the slow grower mycobacteria specific 16S rRNA gene specific primers (APTK16S). L is a reference sizing ladder (TrackIt™ 50 bp DNA Ladder, Invitrogen, Ltd., Paisley, UK), C is the negative PCR control, samples 1–4 are the four Ethiopian soils (1108, 1109, 1110, 1111) and 5 is Cryfield. The arrows (2A–2I) represent the bands that were excised and sequenced (Table 4).

Resolution of EM at the species level with DGGE was achieved using both genus specific and slow grower only targets (Fig. 1). Optimization with clinical isolates singly and as equimolar mixtures was performed prior to profiling of soil indigenous EM communities (Figure 1A, B). The optimized protocol was then used for comparative analysis of the total soil bacterial community (Fig. 1 C and D). A range of DGGE bands were sequenced (Table 4). Genus specific DGGE bands yielded predominantly fast growers (>97% sequence similarity) (DGGE-1A and DGGE-1I) whereas slow growers specific DGGE yielded exclusively slow growing mycobacteria (DGGE-2A and DGGE-2I). All of the genus and slow growers DGGE blast matches apart from one *(M. haemophilum)* were also identified in the pyrosequencing data for the corresponding samples. It was expected that the DGGE bands would represent the most prevalent species present in the sample, as judged by pyrosequencing, as it was the case for *M. moriokaense* in sample 1110 and *M. colombiense* in samples 1108 and 1109. However, this was not always the case; for example, in the Cryfield sample *M. sp. JS623* and *M. sp. GR-2001-270* only represented a small proportion of the pyrosequencing reads. This suggests that DGGE bands may not consistently represent the most abundant species, but that the species identified are likely to be present in the sample as shown by the pyrosequencing.

**Table 4:** Sequence comparisons of bands excised from DGGE gels (Fig. 1 C and D) and blasted against the *16S rRNA* gene database retrieved from SILVA.

*Mycobacterium* genus specific primers (JSY16S) DGGE					Slow growing *Mycobacteria* specific primers (APTK16Slow) DGGE				
DGGE band	Identity (%)	E value	Match accession number	Match name	DGGE band	Identity (%)	E value	Match accession number	Match name
1A	98.08	0	DQ249999	*Mycobacterium sp.* L47	2A	99.42	0	GQ153275	*Mycobacterium colombiense*
1B	99.73	0	EU165538	*Mycobacterium brasiliensis*	2B	97.28	3×10^−168^	GQ153275	*Mycobacterium colombiense*
1C	99.44	0	X93029	*Mycobacterium sp.*	2C	99.71	0	FJ794352	*Mycobacterium sp.* NLA000202017
1D	100	2×10^−64^	EF019937	Uncultured *Mycobacteriaceae bacterium*	2D	99.71	0	GQ153275	*Mycobacterium colombiense*
1E	97.36	0	AY859686	*Mycobacterium moriokaense*	2E	99.71	0	GQ153275	*Mycobacterium colombiense*
1F	98.91	0	AY859686	*Mycobacterium moriokaense*	2F	99.13	0	EU274642	*Mycobacterium riyadhense*
1G	98.52	0	FJ605266	*Mycobacterium sp.* DCY42	2G	99.09	0	EU274642	*Mycobacterium riyadhense*
1H	98.31	0	AY162028	*Mycobacterium sp.* JS623	2H	99.71	0	GQ153275	*Mycobacterium colombiense*
1I	97.53	4×10^−145^	FJ538898	*Mycobacterium sp.* GR-2001-270	2I	98.85	0	U06638	*Mycobacterium haemophilum*

### OTU based diversity analysis of EM

**Figure 2:**
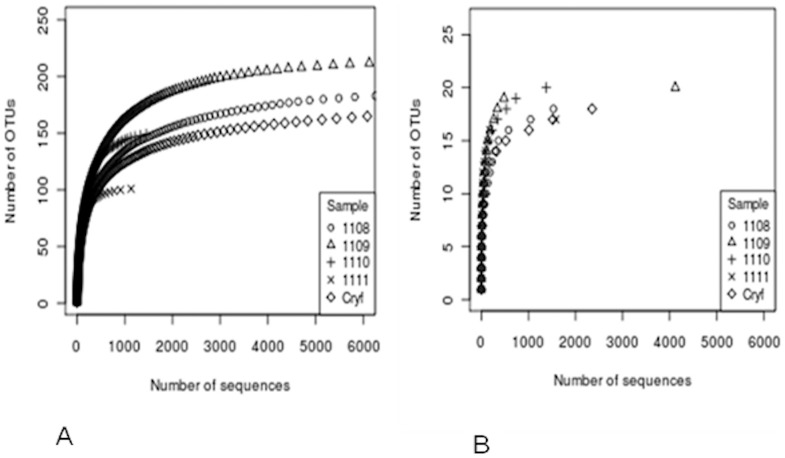
OTU based diversity analysis. Rarefaction curves for EM (A) and slow growing EM (B) data sets.

Five soil samples were deep sequenced with two primer sets targeting the EM genus and slow growers only. A total of 27258 (genus specific) and 10970 (slow growers specific) reads were retained for analysis post quality control (Table 3). The number of reads of individual samples after trimming was in the range of 1000 to 9000 for the genus specific data set and 1000 to 4000 for the slow growers data set. The number of clusters obtained after binning with CD-HIT at the cutoff of 97% similarity is also given (Table 3). Binning was also done at the 99% cutoff. As an example, for the slow growing EM dataset, the more stringent binning cutoff revealed a higher number of clusters (336 versus 33). Notwithstanding the higher number of clusters, in reality this only corresponded to a few additional species obtained as compared to those obtained with the 97% id clustering cutoff (i.e. 24 taxonomical identities for 335 clusters against 15 identities with 33 clusters). The rarefaction curves (Fig. 2) indicated that the sampling effort was adequate with both primers sets.

With the genus specific primers, a total of 314 clusters or OTUs were identified (Table 3 and Table S1 in File S1) across the five sites. This represented a ten-fold higher figure than the number of OTUs obtained with the slow grower data set. However, only 177 (56%) of these OTUs contained more than 10 sequences per cluster and there were 66 singleton clusters (21%), which suggests either high diversity of rare *Mycobacterium* species, or potential sequencing errors which is considered less likely due to the quality controls adopted for culling chimeras and sequences with ambigous base calls.

After phylogenetic assignment using BLASTn, 88% of the data set (279 clusters) corresponded to 96 species that were culturable or clinical isolates, the remaining 35 clusters were matched to uncultured or unidentified species. However, only 153 clusters (50%), representing 86% of the sequences (23396), could be assigned to known species at the 97% similarity cutoff. All sequences could be assigned to the *Mycobacterium* genus by classifying the sequences against the Silva *Mycobacterium* database (overall, identities had ranged from 92 to 100%, with the cutoff of 97% used to retain species). Only 60% ca. of these 153 clusters, corresponding to 29 species, had a prevalence higher than >1% (Table S1 in File S1). After classification with BLASTn to the reference *Mycobacterium* database, the top ten species identified matched to *uncultured Mycobacterium sp.* (5.3%), *M. chlorophenolicum* (2 clusters, 4.7%); *M. sp. GR 2009-164* (4.2%); *M. neglectum* (4 clusters, 3.5%); *M. vaccae* (3.28%); *M. holsaticum* (3.2%) *M. moriokaense* (2.27%), *Mycobacterium sp*. T126 (2.2%) and *Mycobacterium sp. DCY42* (2.1%). These species were originally isolated from a variety of samples including clinical material (*M. sp. GR 2009-164, M. vaccae, M. holsaticum*), soil (*M. moriokaense*, *M. chlorophenolicum, M. sp*. T126 and *M. sp. DCY42, M. vaccae*) and water (*M. neglectum*). For the genus data set, chimeras were found respectively in cluster 4, *M. vaccae* (482/896 seqs), cluster 7, *M. moriokaense* (56/607 seqs), cluster 45, *Mycobacterium sp. Ellin148* (48/606 seqs), cluster 125, *Mycobacterium sp. WF2* (51/1150 seqs), cluster 149, *M. sp GR- 2009-164* (101/1150 seqs), cluster 167, *Mycobacterium sp. WF2* (111/145 seqs).

For the slow grower data set, a total of 33 representative clusters were identified across five soils (Table 3, and Table S2 in File S1). Of these, four were singletons, 11 were unique clusters to one soil, whilst the remaining 22 were shared between the five soil samples. Excluding the singletons, only 11 clusters had a prevalence higher than 1.5%. About 99% of the 10970 (9941 reads) were matched to a sequence in the Silva database with a similarity higher than 97%; those below this cutoff were excluded from the analysis. These sequences were classified (Table S2 in File S1) as *M. colombiense* (7 clusters, which represented 40% of the total) and *M. gordonae* (8 clusters, 13%), *M. aemonae* (1 cluster, 13%), *M. asiaticum* (4 clusters, 11% of the total), *M. malmoense* (2 clusters, 8% of the total), *M. riyadhense*, (1 cluster, 7%) *M. sp. NLA000202017* (1 cluster, 2.75%) and *M. angelicum* (2 clusters, 1.4%). The predominant species listed above were often represented by several clusters, but generally only a few of these contained the majority of sequences, this was the case for *M. colombiense* where Cluster 4 was the most conspicous (4153 sequences, 38.13% of the total). Interestingly, only sequences from the Cryfield site were identified as *M. tuberculosis* complex in Cluster 14, but these had very low prevalence (0.1% of the total). For the slow grower data set chimeras were predominantly found in cluster 13, *M. angelicum* (29/142), cluster 3, *M. aemonae* (18/1519) and cluster 0, *M. riyadhense* (7/760).

### Diversity metrics of EM

Diversity indices Shannon (H') and Simpson varied considerably between the genus and slow grower sequences. For the genus data set, they were generally higher than with the slow growers data set (Table 3), reflecting that the diversity attained was higher when the whole genus was targeted.

Considering the diversity estimators CHAO1 and ACE, a χ^2^ test between the number of expected sequences and those observed, revealed that the sequencing effort was adequate for the slow grower data set (p>0.05). For the genus specific primer set, however, there were statistically significant differences (p<0.05) between the number of unique OTU observed and those expected, indicating that the EM diversity for these soil samples was not fully explored, although the rarefaction curves had plateaued (Fig. 2 A).

### Random sequence resampling

For any biological system, there is an intrinsic variability between samples, with uneveness being introduced during the sampling and during the downstream molecular assays. In this case the number of reads obtained for each sample varied considerably between samples thought to be due to amplification biases, inhibitory compounds carried over in the soil extracts, or the stochasticity of sequencing reactions. Therefore, randomised resampling was also performed in order to remove any potential bias in the computation of the indices (Table 3) and to assess the reliability of the bioinformatics approach. The genus data set size was normalized to 1135 sequences and the slow grower data set to 1381 sequences. When controlling for sequencing effort, no statistical differences were observed in the number of clusters, the diversity indices H' or Simpson.

### Standalone blast of the sequences

**Figure 3:**
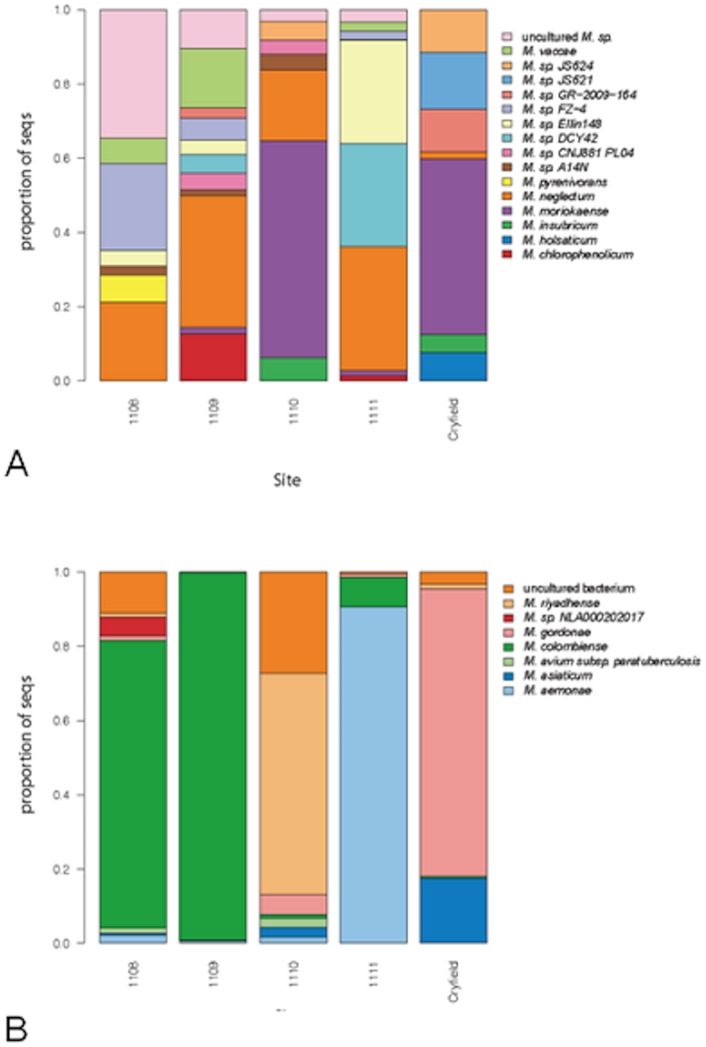
Visual representation of the beta diversity across sites. Proportion (%) of species in each sample after blasting the whole data sets containing the amplicons obtained with genus (A) and slow grower (B) specific primer sets.

Independently from the results obtained with the taxon based approach with clustering sequences into OTUs, entire data sets were also compared to the same blast databases, with only sequences having similarity equal or higher than 97% retained. A comparison of diversity of EM within soils, revealed site specific assemblages of mycobacteria (Fig. 3A and 3B). Overall, 16 species for the genus data set and 7 species for the slow growing EM data set were >1% prevalent.

It is true to say that the genus data set predominantly harboured previously described species (compared with the OTU based diversity analysis) with the remaining sequences that were similar to those originally isolated from a variety of sources including chlorinated contaminated sites or activated sludge, marine and riverbed sediment or soil. Cryfield site uniquely harbored species *Mycobacterium sp.* JS621 and *M. holsaticum*, a clinical isolate. In common with Ethiopian sample 1110, the Cryfield soil also contained a high proportion of *M. moriokaense,* a soil dwelling microbe. Only the Ethiopian samples possessed *M. vaccae* and *M. cholorophenolicum*. They also contained the majority of *M. neglectum*, originally isolated from drinking water biofilm. Examining the slow grower data set revealed clear differences across sample types with the Ethiopian samples clearly showing distinct compositions compared to the Cryfield. Ethiopian samples 1108 and 1109 were dominated by *M. colombiense*, which was absent from Cryfield. Sample 1108 also harboured *M. sp. NLA00020202017*, which is also a member of the *M. avium* complex as indeed is *M. colombiense*. *M. aemonae* and *M. riyadhense* were only present in the Ethiopian samples whilst *M. gordonae* and *M. asiaticum* were unique to Cryfield.

A comparison of the clustering approach with the the taxon independent approach, i.e. blasting the clusters to the same database used for the standalone blast of the sole sequences, revealed that the same species were found with both approaches, reinforcing the validity of these independent methods.

### Linking OTUs to phylogeny

**Figure 4:**
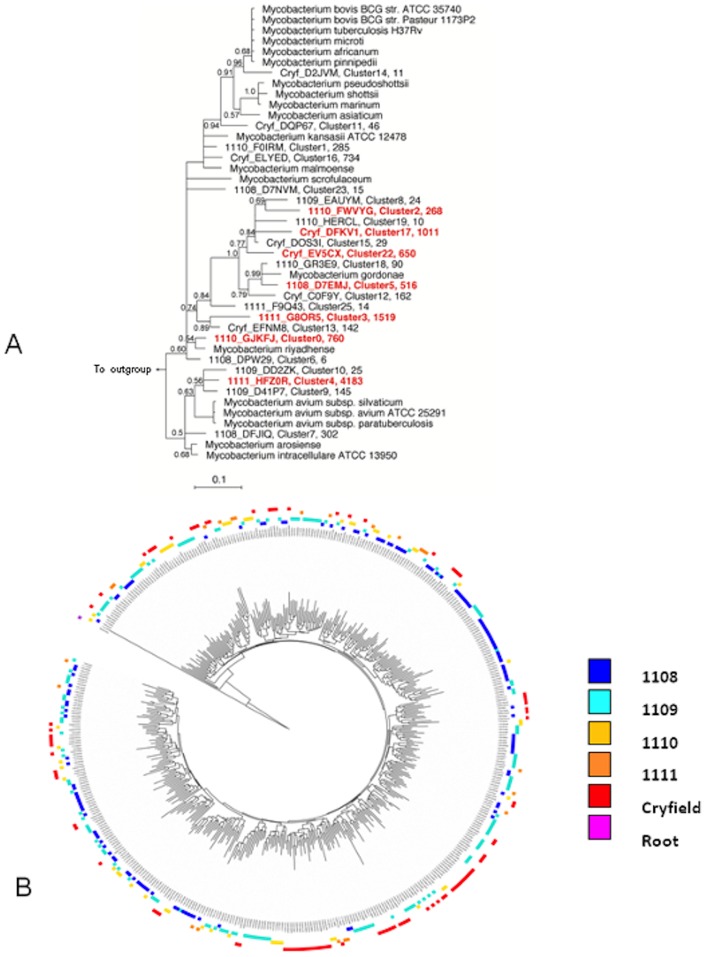
Phylogeny based analysis. (A) Phylogenetic position of the 33 representative OTU (with the original sequence tag, the cluster number and the total number of sequences) after binning with CD HIT EST at the 3% cutoff the sequences obtained with the APTK16S primer. Predominant clusters are highlighted in red. The tree was made with MrBayes with the PhymL maximum likelihood algorithm and includes a non-redundant list of the best matches of each sequence as defined by the Silva project (Release 104). (B) ITOL drawn tree containing 314 representative sequences for each OTU obtained with the genus specific primer set defined showing topology of sites in the outer ring (see site colour coding) and significant phylogenetic clustering as indicated by the FastUnifrac P test significance (P<0.01).

For both data sets, when the reference sequences representing the various clusters were aligned to the reference set of mycobacterial sequences and imported into ARB, their position on the tree correlated well with the independent result of the standalone blast analysis (Fig. 4A). For reasons of brevity, only the tree relative to the slow data set is shown, with clusters with the highest prevalence labeled in red.

### Phylogenetic diversity analysis

Unweighted FastUnifrac was used to compare the diversity of microbial communities using a phylogenetic based approach. The distribution of sequences was significantly different between sites regardless of whether all samples were analyzed together, each sample individually or with pairwise analysis. For both the genus level and the slow grower data set, the distance metrics indicated that branch lengths across samples were significantly unique and not due to chance (uncorrected p<0.0001, corrected p<0.002). Significant differences (p<0.002) between each pair of samples were observed after the Bonferroni correction for multiple comparisons. The exception being for the slow growers data set, where if the samples were compared individually, sample distance p values for sample 1109 indicated that the tree topology for this site was not significantly unique. The P test within the FastUnifrac package, which reveals if samples are significantly clustered on the tree, was also performed for both data sets. For the genus data set, when comparing each pair of samples, clustering was highly significant (raw p<0.001 and after the Bonferroni correction for multiple comparisons, p<0.002). When all samples were analysed together (unweighted mode, 500 permutations) again clustering was significant (p<0.01) (qualitatively illustrated in Fig. 4 B). For the slow data set this test gave similar results to the genus level. Overall, the comparative phylogenetic analysis suggests that EM communities are significantly different in the five soil samples.

### Analytical sensitivity of DGGE method

The analytical sensitivity with the genus specific JSY16S primer set was 10^5^ BCG gene targets g^−1^ of soil; nested PCR did not improve the limit of detection. When the slow grower specific APTK16S was used, the detection limit was 10^3^ gene targets g^−1^ of soil which improved to 10^2^ BCG gene targets g^−1^ of soil with the nested approach.

## Discussion

This paper reports a new approach devised to detect and quantify EM in soil, using a combination of qPCR, DGGE and Pyrosequencing. To our knowledge, this is the first paper to report analysis of EM diversity using the same target gene, whilst attempting to distinguish slow growers from the rest of the mycobacteria. We report successful testing and validation of these methods for the detection of EM in two very distinct sites.

Novel primers were designed and optimized to detect slow growing mycobacteria, and previously designed genus specific primers were validated with two of the three methods and optimized for qPCR. We were able to segregate slow and fast growing members of the *Mycobacterium* genus and, as expected, fast growers were more prevalent in all soils comparing all three methods. Our data shows that the prevalence of EM as tested by qPCR is consistent with what has been reported for the genus: using a qPCR assay targeting a different region of the rRNA operon, cell counts ranged from 10^4^ to 10^7^ cells g^−1^ of house dust [29] and [30].

High-throughput pyrosequencing offers an opportunity to study in depth the diversity of mycobacteria in soil. Two complementary approaches were taken with the analyses, an OTU based and a phylogeny based method. A comparison of the clustering approach with the the taxon independent approach, i.e. blasting the clusters to the same database used for the standalone blast of the sole sequences, revealed that the same species were found with both approaches, reinforcing the validity of these independent methods. A number of points regarding the optimisation of the pyrosequencing merit discussion.

A 97% binning cutoff was chosen – analysis was also done at a more stringent clustering cutoff of 99% but this did not significantly alter the most preponderant species identities found or the overall numbers of sequences affiliated to these.

The less stringent cutoff of 97% was selected as the conservative choice given the demonstrated errors in pyrosequencing [39] and so it was used for the OTU based analysis. CD-HIT-EST is a powerful clustering tool which enables management of large amount of data with a reduced set of representative sequences and downstream diversity analysis. However, in our case, where the target was a specific genus and a subdivision within a genus, this approach resulted in some very similar clusters; hence the subsequent diversity analysis is very likely overestimating diversity due to inflated numbers of OTUs. Similarly, for the slow growing EM dataset, a binning cutoff at 99% against 97% increased the number of the OTU by ten-fold without significantly increasing the number of exact species matches.

Using universal primers for pyrosequencing, the proportion of sequences that belonged to the *Mycobacterium* genus in the soil varied from 0.49% to 2.3% of the total reads (S. Dowd pers. comm.) which indicated a significant numerical presence of EM in the environment, which was also confirmed in our study by qPCR.

Determining the exact prevalence of EM load within the environment required a molecular approach and was achieved by dual focus on both fast and slow growing mycobacteria as the latter are difficult to detect due to higher prevalence of fast growers, and because we can cultivate only a very small proportion of the estimated 10^10^ bacterial species [49] contained within a gram of soil. Moreover, cultivation-based approaches are well known to underestimate and bias the number of species by orders of magnitude [50].

Although different regions of the 16S rRNA gene were targeted for the genus specific and slow grower analysis, our work supports indeed the hypothesis that the number of EM species in the environment is higher than the number of species that has been isolated to date. Pyrosequencing indicated more unknown groups within the genus data set with most of the new diversity grouping with fast growers (14%), whereas about only 1% of sequences in the slow grower data set did not match known identities in the database. In addition, for the genus data set only, the Chao1 and ACE statistics suggested that greater diversity could be expected to be recovered with additional sequencing.

In general, the DGGE profiles correlated well with the pyrosequencing approach, detecting the most abundant species, particularly for the slow growers' data set. This included *M. colombiense*, a recently isolated opportunistic pathogen [51], and *M. riyadhense*, previously related to the *M. tuberculosis* group based on biochemical tests, but recently separated based on phylogeny [52].

In fact, only one slow grower DGGE band (*M. haemophilum* from Cryfield soil) was not also identified through pyrosequencing. For the genus data set there was a fairly good correlation between the methods in some cases. For example, *M. moriokaense* was the most abundant species detected in both DGGE and pyrosequencing for sample 1110. However, prevalent sequences in pyrosequenced libraries did not always correspond to prevalent DGGE bands. This suggests that DGGE bands may not consistently represent the most abundant species, but that the species identified are likely to be present in the sample as shown by the pyrosequencing. However pyrosequencing has its own amplification biases as for instance those due to the variability in the EM content across different technical replicates. Differences in PCR amplification including a preliminary round of nesting may have been the causes of minor discrepancies observed between DGGE and pyrosequencing. In addition, DGGE may not be as robust as deep sequencing for inferring beta diversity correlations within the genus, as illustrated by two bands matching to *M. moriokaense* that were isolated from the DGGE gel at different positions. Overall, DGGE has its well documented drawbacks [53], but in our case it has proved, with new and customized primers for EM, to provide a reliable snapshot of the environmental microbial diversity, as previously shown in our lab [54].

Slow growing bacteria present a considerable challenge for community analysis and thus a molecular approach is currently the only feasible option for studying slow growing mycobacterial reservoirs in the environment. However, caveats must be considered due to the well-known biases introduced by PCR based methodology, which is common to all the three methods employed in this study. In our work, in order to control for the unevenness of the reads across samples, a random resample of the sequences was also performed to test the reproducibility of the data from the OTU dependent approach. Although some variation could be observed and anticipated, overall the differences in the diversity indexes of the original and the resampled data sets were not significant, with higher diversity still being observed for the genus data set. Whilst there are other drawbacks in PCR based approaches [55], [56], these are at present the only sufficiently valid methods to estimate diversity and prevalence of target genera in the environment.

In this study, we attempted identification of *Mycobacterium* to the species level using several approaches, all based on the 16S rRNA genes which are commonly used for phylogenetic studies concerning the genus *Mycobacterium,* in particular the first 500 bp, which include its major hypervariable regions ([57]; [5]; [31]). However, a non-negligible limit of such phylogenetic reconstructions is the poor robustness of the trees. A 97% cutoff for species level discrimination comparisons to a comprehensive reference database is also a globally accepted procedure. However, a 97% species identity cut off may not properly resolve some species complexes such as *M. tuberculosis* and *M. avium* or individuals exceptionally close to the *M. tuberculosis* group such as *M. lacus, M. marinum and M. ulcerans* for the slow growers specific target region and for a few fast growers such as *M. szulgai* (Tables S3 and S4 in File S1) for the genus specific region.

To improve phylogenetic accuracy and robustness, more recently the multilocus approach [58] has been used in two large studies ([59]; [60]) in which four and seven different nucleotide fragments, respectively, were concatenated. These approaches are useful, but are not yet applicable for culture independent high throughput methods for environmental surveys of mycobacteria as reported here [61]. More recent studies based on datasets obtained with next generation sequencing technologies, which report results from the Human Microbiome Project [62] and species level analysis of the latter datasets [63] show that accurate species labeling is dependent on the choice of the region. For instance, for the *Staphylococcus* genus and, globally, for the larger HMP dataset the V1–V3 region had a higher species resolving power, and this is the same target region for the analysis of the slow growers EM in this study.

The approach described here can now be used for larger scale environmental surveys devoted to establishing if EM prevalence and diversity and human exposure to these EM could be linked to the TB infectious status at different geographical settings, and what role these conditions play in the reduction of the BCG vaccine efficacy at southern latitudes. In conclusion, we report here of the successful use of PCR based methodology employing two primer sets on the metagenome of five example soils with varying characteristics. These methods revealed with an unprecedented level of resolution the prevalence and diversity of EM in soil.

## Supporting Information

File S1 Table S1, Clusters characteristics for the genus data set. Only representative sequences with a prevalence higher than 1% are displayed, alongside with the information related to n° of sequences per cluster, the relative proportion and the sequence accession number after BLASTn analysis. Table S2, Clusters characteristics for the slow growing EM data set. Representative sequences are displayed alongside with the information related to the n° of sequences per cluster, the relative proportion and the sequence accession number after BLASTn analysis. Table S3, Sequence difference count matrix for 21 slow growing strains as compared to 51 slow and fast growing EM for theAPTK16S primers target region (slow growing EM specific). ID = identical strains. Table S4, Sequence difference count matrix for 51 EM strains for the JSY16S primers target region (*Mycobacterium* genus specific). ID = identical strains.
(DOCX)
