# Supplementary material for: Prospecting Environmental Mycobacteria: Combined Molecular Approaches Reveal Unprecedented Diversity
Source: PLoS One. 2013 Jul 18;8(7):e68648. doi: 10.1371/journal.pone.0068648 (PMC3715504; doi:10.1371/journal.pone.0068648)
Supplement: File S1 — Table S1, Clusters characteristics for the genus data set. Only representative sequences with a prevalence higher than 1% are displayed, alongside with the information related to n° of sequences per cluster, the relative proportion and the sequence accession number after BLASTn analysis. Table S2, Clusters characteristics for the slow growing EM data set. Representative sequences are displayed alongside with the information related to the n° of sequences per cluster, the relative proportion and the sequence accession number after BLASTn analysis. Table S3, Sequence difference count matrix for 21 slow growing strains as compared to 51 slow and fast growing EM for theAPTK16S primers target region (slow growing EM specific). ID = identical strains. Table S4, Sequence difference count matrix for 51 EM strains for the JSY16S primers target region (Mycobacterium genus specific). ID = identical strains. (DOCX) [file pone.0068648.s001.docx]

| seq_id | Cluster n° | Num. seqs | Prop.seqs | Species name | accession n° |
| --- | --- | --- | --- | --- | --- |
|  |  |  |  |  |  |
| 1109_DAIUW | 53 | 648 | 2.3773% | *Mycobacterium chlorophenolicum* | X79292 |
| 1110_HDX2W | 112 | 310 | 1.1373% | *Mycobacterium coloregonium* | AY624367 |
| 1108_DIFS0 | 3 | 395 | 1.4491% | *Mycobacterium conceptionense* | GQ342298 |
| Cryfield_EQKEM | 83 | 874 | 3.2064% | *Mycobacterium holsaticum* | AJ310467 |
| Cryfield_ESRZU | 233 | 323 | 1.1850% | *Mycobacterium holsaticum* | AJ310467 |
| 1110_HJ0NZ | 161 | 403 | 1.4785% | *Mycobacterium houstonense* | EU977810 |
| 1108_C1LLA | 7 | 607 | 2.2269% | *Mycobacterium moriokaense* | AY859686 |
| Cryfield_DZP48 | 189 | 348 | 1.2767% | *Mycobacterium moriokaense* | AY859686 |
| 1110_HA1XO | 114 | 955 | 3.5036% | *Mycobacterium neglectum* | AJ580802 |
| 1108_DOE1P | 0 | 641 | 2.3516% | *Mycobacterium neglectum* | AJ580802 |
| 1109_EF3BL | 49 | 411 | 1.5078% | *Mycobacterium neglectum* | AJ580802 |
| 1109_EPMC9 | 69 | 373 | 1.3684% | *Mycobacterium neglectum* | AJ580802 |
| 1108_EFF8I | 10 | 462 | 1.6949% | *Mycobacterium pyrenivorans* | AJ431371 |
| 1108_C22R2 | 14 | 528 | 1.9370% | *Mycobacterium sp. CNJ881 PL04* | DQ448781 |
| 1109_EU91M | 62 | 574 | 2.1058% | *Mycobacterium sp. DCY42* | FJ605266 |
| Cryfield_EQKTD | 104 | 377 | 1.3831% | *Mycobacterium sp. DSM 3803* | AY147261 |
| 1108_ENQ3T | 16 | 350 | 1.2840% | *Mycobacterium sp. Ellin113* | AF408955 |
| 1109_CZR5J | 54 | 512 | 1.8783% | *Mycobacterium sp. Ellin182* | AF409024 |
| Cryfield_DKK1N | 149 | 1150 | 4.2189% | *Mycobacterium sp. GR-2009-164* | FJ555540 |
| Cryfield_EW4KS | 103 | 488 | 1.7903% | *Mycobacterium sp. JS623* | AY162028 |
| 1109_EW6MW | 47 | 477 | 1.7499% | *Mycobacterium sp. JS624* | AY162029 |
| 1110_GUB6L | 116 | 293 | 1.0749% | *Mycobacterium sp. JS624* | AY162029 |
| Cryfield_D27MP | 98 | 340 | 1.2473% | *Mycobacterium sp. KAR6* | EF451636 |
| 1108_EGAQT | 45 | 606 | 2.2232% | *Mycobacterium sp. T126* | FJ719354 |
| 1109_DZIGM | 78 | 371 | 1.3611% | *Mycobacterium tusciae* | AF058299 |
| 1108_C942V | 4 | 896 | 3.2871% | *Mycobacterium vaccae* | AF544638 |
| 1108_D3QDW | 6 | 333 | 1.2217% | *uncultured Mycobacteriaceae* | EF019277 |
| 1108_EKDHQ | 24 | 1449 | 5.3159% | *uncultured Mycobacterium sp.* | FJ542897 |
| 1108_DIHVQ | 34 | 277 | 1.0162% | *uncultured Mycobacterium sp* | FJ542897 |

**Table S1**.

| seq_id | Cluster n° | Num. seqs | Prop. seqs | Species name | accession n° |
| --- | --- | --- | --- | --- | --- |
|  |  |  |  |  |  |
| 1111_G8OR5 | Cluster 3 | 1519 | 13.8469% | *Mycobacterium aemonae* | FJ794352 |
| 1108_DPW29 | Cluster 6 | 6 | 0.0547% | *Mycobacterium angelicum* | AM884328 |
| Cryf_EFNM8 | Cluster 13 | 142 | 1.2944% | *Mycobacterium angelicum* | GQ153277 |
| 1110_GJTYR | Cluster 24 | 5 | 0.0456% | *Mycobacterium asiaticum* | EF428556 |
| 1110_HERCL | Cluster 19 | 10 | 0.0912% | *Mycobacterium asiaticum* | EF428556 |
| 1111_F9Q43 | Cluster 25 | 14 | 0.1276% | *Mycobacterium asiaticum* | GQ153275 |
| Cryf_DFKV1 | Cluster 17 | 1011 | 9.2160% | *Mycobacterium asiaticum* | AF480595 |
| 1110_GRVIL | Cluster 31 | 1 | 0.0091% | *M. avium subsp. paratuberculosis* | GQ153278 |
| 1109_EY4NC | Cluster 32 | 1 | 0.0091% | *Mycobacterium colombiense* | AM884328 |
| 1109_DIX0Z | Cluster 27 | 2 | 0.0182% | *Mycobacterium colombiense* | GQ153275 |
| 1109_EM8U7 | Cluster 28 | 2 | 0.0182% | *Mycobacterium colombiense* | AM884330 |
| 1109_C33JP | Cluster 29 | 3 | 0.0273% | *Mycobacterium colombiense* | AY604571 |
| 1109_DD2ZK | Cluster 10 | 25 | 0.2279% | *Mycobacterium colombiense* | AF480595 |
| 1109_D41P7 | Cluster 9 | 145 | 1.3218% | *Mycobacterium colombiense* | X58890 |
| 1111_HFZ0R | Cluster 4 | 4183 | 38.1313% | *Mycobacterium colombiense* | AM935142 |
| 1110_FZ7QO | Cluster 30 | 2 | 0.0182% | *Mycobacterium gordonae* | GQ153278 |
| 1109_EAUYM | Cluster 8 | 24 | 0.2188% | *Mycobacterium gordonae* | EF428556 |
| Cryf_DOS3I | Cluster 15 | 29 | 0.2644% | *Mycobacterium gordonae* | AF480595 |
| 1110_GR3E9 | Cluster 18 | 90 | 0.8204% | *Mycobacterium gordonae* | GQ153275 |
| Cryf_C0F9Y | Cluster 12 | 162 | 1.4768% | *Mycobacterium gordonae* | GQ153275 |
| 1110_FWVYG | Cluster 2 | 268 | 2.4430% | *Mycobacterium gordonae* | AY652958 |
| 1108_D7EMJ | Cluster 5 | 516 | 4.7037% | *Mycobacterium gordonae* | X52934 |
| Cryf_EV5CX | Cluster 22 | 650 | 5.9253% | *Mycobacterium gordonae* | GQ153275 |
| 1109_D2ZA0 | Cluster 21 | 1 | 0.0091% | *Mycobacterium intracellulare* | DQ536404 |
| 1110_F0IRM | Cluster 1 | 285 | 2.5980% | *Mycobacterium malmoense* | GQ153275 |
| Cryf_ELYED | Cluster 16 | 734 | 6.6910% | *Mycobacterium malmoense* | EF428556 |
| Cryf_DQP67 | Cluster 11 | 46 | 0.4193% | *Mycobacterium nebraskense* | EF428556 |
| 1110_GJKFJ | Cluster 0 | 760 | 6.9280% | *Mycobacterium riyadhense* | AF480595 |
| 1108_DMODA | Cluster 20 | 5 | 0.0456% | *Mycobacterium salmoniphilum* | DQ866767 |
| 1108_D7NVM | Cluster 23 | 15 | 0.1367% | *Mycobacterium sp. 31181* | EU274642 |
| 1108_DFJIQ | Cluster 7 | 302 | 2.7530% | *Mycobacterium sp. NLA000202017* | EF428556 |
| Cryf_D2JVM | Cluster 14 | 11 | 0.1003% | *Mycobacterium tuberculosis* | EF428556 |
| 1111_FP5ZG | Cluster 26 | 1 | 0.0091% | *uncultured Mycobacterium sp.* | GQ153275 |

**Table S2**.

**Table S3.**

 **Table S4.**
